# Meta analysis of the effect of phloroglucinol combined with progesterone in the treatment of threatened miscarriage before 20 weeks of gestation: A protocol for a systematic review

**DOI:** 10.1097/MD.0000000000031885

**Published:** 2022-11-25

**Authors:** Yao Peng, Jiayuan Zhang, Tian Lan, Shengyue Liu, Tao Ye, Yongzhou Wang

**Affiliations:** a The Affiliated TCM Hospital of Southwest Medical University, Luzhou, China; b Sichuan Police College, Luzhou, China.

**Keywords:** meta-analysis, phloroglucinol, progesterone, threatened miscarriage

## Abstract

Threatened miscarriage (TM) is an important factor endangering the health of pregnant women. It not only affects women’s physical and mental health, but also destroys family happiness. To treat this disease, it is necessary to find a treatment with better clinical efficacy and fewer side effects. The purpose of this systematic study was to evaluate the efficacy and safety of phloroglucinol (PHL) combined with progesterone in the treatment of TM before 20 weeks of pregnancy.

**Methods:**

Electronic databases (EMBASE, PubMed, Cochrane Central Register of Controlled Trials, Web of Science, Elsevier, China National Knowledge Infrastructure, Chongqing VIP, and WanFang Data) were searched from inception until September. 2022. Randomized controlled trials of PHL combined with progesterone in the treatment of TM before 20 weeks of gestation will be included, and all articles will be independently screened and collected by 2 reviewers. Revman 5.3.5 software will be used for meta-analysis. The specific process is described in the Cochrane Handbook for Systematic Reviews.

**Results:**

The efficacy and safety of PHL combined with progesterone for the treatment of threatened abortion were comprehensively evaluated in terms of efficacy, efficiency, time of symptom relief, length of hospital stay, and incidence of adverse events.

**Conclusion:**

This study provides reliable evidence for the clinical application of PHL combined with progesterone for the treatment of TM.

## 1. Introduction

Threatened miscarriage (TM) refers to a small amount of vaginal bleeding, paroxysmal lower abdominal pain, or low back pain before 28 weeks of pregnancy, with an unopened uterine orifice, intact fetal membrane, and fetal survival.^[1]^ With the rapid development of society, the change of lifestyle, and the increasingly serious environmental pollution, the incidence of threatened abortion is increasing.^[2]^ Some research shows that the rate of threatened abortion in pregnant women is as high as 30% to 40%. Most pregnant women can continue their pregnancy after rest and treatment. However, there are still some pregnant women whose symptoms cannot be alleviated, which eventually leads to abortion.^[3]^ If not treated in time, it is easy to develop into an inevitable or incomplete miscarriage, causing infection or even shock, which seriously threatens the life of women during pregnancy.^[4,5]^ At present, the clinical treatment of TM mainly uses progestogen products, dydrogesterone belongs to the progestogen drugs, is the treatment of early threatened abortion drugs commonly used, and can increase the level of pregnant women’s blood progesterone.^[6–8]^ However, there are some disputes on the medical treatment of threatened abortion. For example, there is no accurate and appropriate method to judge the luteal function, and there are also differences in whether progesterone injection is effective.^[9–13]^ Phloroglucinol (PHL), as a mesophilic non-atropine and non-papaverine drug, is a trinitrobenzene derivative with an antispasmodic effect.^[14]^ Since the 1960s, it has been used for various diseases.^[15]^ Because PHL has a rapid effect^[16]^ and few adverse reactions,^[17,18]^ it has been widely used in the department of gynecology, obstetrics, and assisted reproduction.

However, the clinical efficacy of PHL combined with progesterone in the treatment of TM lacks systematic evaluation of evidence-based medicine. In the present study, a meta-analysis was performed to comprehensively evaluate the efficacy and safety of PHL combined with progesterone in the treatment of TM to provide credible evidence for clinical decision-making for people at risk of miscarriage.

## 2. Methods

### 2.1. Registration

This study has been registered on PROSPERO as CRD42022360203. In this paper, the protocol will be performed using the methods introduced in the Cochrane Handbook for Systematic Reviews of Intervention^[19]^ and reported according to the the preferred reporting items for systematic reviews and meta-analyses (PRISMA)-P guidelines.^[20]^ If we will refine procedures described in this protocol, we will document the amendments in the PROSPERO database and disclose them in future publications related to this meta-analysis.

### 2.2. Inclusion criteria

#### 2.2.1. Types of studies.

Randomized controlled trials regarding PHL combined with progesterone in the treatment of TM in Chinese or English were included, regardless of study blinding. This study is based on the preferred reporting items for systematic reviews and meta-analysis (PRISMA statement).^[21]^

#### 2.2.2. Types of patients.

All pregnant women who present with vaginal bleeding with or without lower abdominal pain during the first 20 weeks gestation, while the cervix is closed and the fetus is viable. There is no limitation on the age, nation of the disease.

#### 2.2.3. Types of interventions.

The experimental group is treated with PHL combined with progesterone, and the control group is treated with conventional progesterone treatment. In addition to intervention measures, other treatment and nursing measures should be consistent between the 2 groups.

### 2.3. Inclusion criteria for outcomes.

#### 2.3.1. Primary outcome.

The total effective rate (i.e., effectual, uterine contractions were obviously relieved or disappeared within 12hours of medication; improved, uterine contractions were relieved to a certain degree within 24hours of medication; and ineffective, symptoms showed no change or were even aggravated within 48hours of medication); the total effective rate = (number of effectual cases + number of improved cases)/ number of total cases 100%.

#### 2.3.2. Secondary outcome.

The rate of adverse reactions; The time to relief of uterine contractions; The complete relief of uterine contraction symptoms; The drug onset time; The duration of the drug treatment; The length of hospital stay.

### 2.4. Data search strategy.

#### 2.4.1. Data sources and searches.

Electronic databases (EMBASE, PubMed, the Cochrane Central Register of Controlled Trials, Web of Science, Elsevier, China National Knowledge Infrastructure, Chongqing VIP, and WanFang Database) are searched from inception until September.2022. There is no restriction on the language of publication.

The search keywords used include the following: “pregnancy,” “gestation,” “fetation,” “PHL,” “phloroglucin,” “progesterone,” “dydrogesterone,” “miscarriage,” “abortion,” “TM,” “threatened abortion,” “randomized controlled trial,” “randomized clinical trial.”

#### 2.4.2. Data extraction and management.

**Figure 1.**
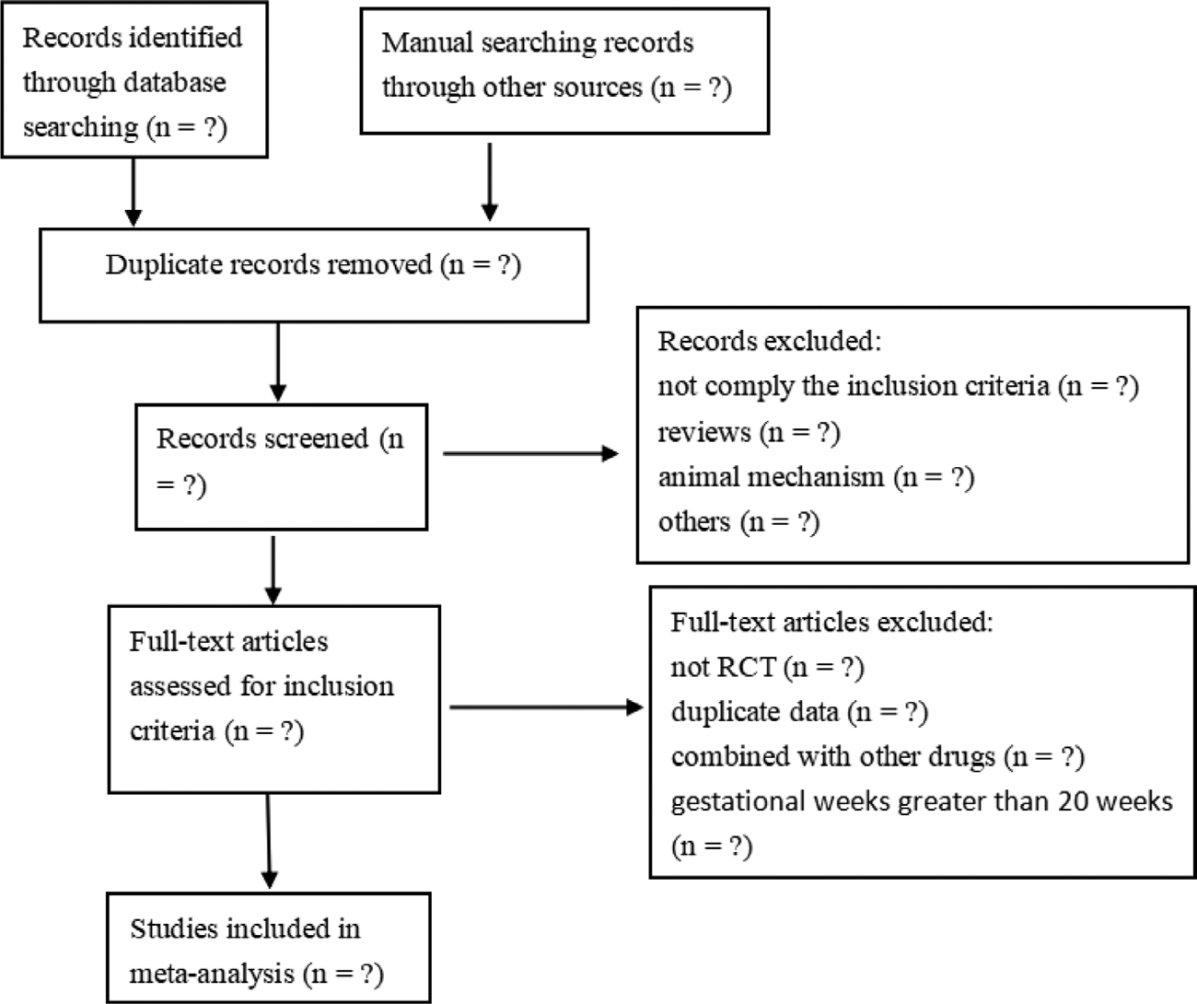
Flow of literature screening.

Literature screening, study selection, and data extraction were performed by 2 reviewers (Yao Peng, Jiayuan Zhang). The literature searched from the electronic database will be imported NoteExpress 3.7 for further screening of the title and abstract, the duplications, and studies not meet the inclusion criteria will be excluded. After reading the full text of the remained literature, and discussing it within the group, the final included studies will be determined. We will try to contact the corresponding author when the full text is unavailable. Disagreements were solved by discussion or consulting a third-party arbitrator until a consensus was achieved. The entire process of study selection is performed in the PRISMA flow diagram, as shown in Fig. 1.

#### 2.4.3. Management of missing data.

If possible, we will contact the original author to request to check the data. Only reasonable and correct data can be used in the primary analysis.

### 2.5. Assessment of risk of bias

Two researchers independently used the “Deviation Risk Assessment Tool” recommended in the Cochrane Handbook 5.1.0 to assess the literature quality. Six points that should be evaluated: random allocation, allocation concealment, blinding, incomplete outcome data, selective outcome reporting, and other biases. Disagreement will also be settled by discussion.

### 2.6. Data analysis.

#### 2.6.1. Strategy for data synthesis.

We will take statistical analysis using the Revman V.5.4 software. We will use fixed-effect meta-analysis for combining data where it is reasonable to assume that studies are estimating the same underlying treatment effect: where trials are examining the same intervention, and the trial’s populations and methods are judged sufficiently similar. If there is clinical heterogeneity sufficient to expect that the underlying treatment effects differ between trials, or if substantial statistical heterogeneity is detected, we will use random-effects meta-analysis to produce an overall summary, if an average treatment effect across trials is considered clinically meaningful. The random-effects summary will be treated as the average range of possible treatment effects and we will discuss the clinical implications of treatment effects differing between trials. If the average treatment effect is not clinically meaningful, we will not combine trials.

#### 2.6.2. Assessment of heterogeneity.

Heterogeneity of the data will be assessed by *Q* test and *I*² statistic. The heterogeneity will be deemed as low when *I*²<50%, moderate (50%–75%), high (*I*² > 75%). The cause of the heterogeneity will be analyzed and a subgroup analysis will be performed.

#### 2.6.3. Analysis of subgroups or subsets.

If we identify substantial heterogeneity, we will investigate it using subgroup analyses and sensitivity analyses. We will consider whether an overall summary is meaningful, and if it is, use random-effects analysis to produce it. We plan to carry out the following subgroup analyses. Gestational age: no more than 12 weeks of gestation versus more than 12 weeks of gestation. The dosage used for PHL. Types of progestins: dydrogesterone or progesterone. We will analyze each subgroup about each of the primary outcomes. We will assess subgroup differences by interaction tests available within Revman V.5.4. We will report the results of subgroup analyses quoting the χ2 statistic and *P* value, and the interaction test *I*² value.

#### 2.6.4. Assessment of publication bias

If there are 10 or more studies in the meta-analysis, we will investigate reporting biases (such as publication bias) using funnel plots. We will assess funnel plot asymmetry visually. If asymmetry is suggested by a visual assessment, we will perform exploratory analyses to investigate it.

#### 2.6.5. Sensitivity analysis.

We will conduct sensitivity analysis based on a) trial quality, excluding trials with unclear/high risk of bias for allocation concealment and sequence generation, and b) published and unpublished studies. We will restrict sensitivity analysis to the review’s primary outcomes.

## 3. Discussion

This meta-analysis will comprehensively review the efficacy and safety of PHL combined with progesterone on TM. Whether it can effectively alleviate the symptoms of TM and reduce the occurrence of side effects. At the same time, it will also detail the deficiencies and prospects of this study. The evidence of this meta-analysis may be beneficial to the clinical treatment of TM, as well as the promotion of traditional Chinese medicine and the formulation of clinical guidelines. However, the differences in specific treatment regimens and methodological quality in each trial can lead to significant heterogeneity.

## Author contributions

**Conceptualization:** Yao Peng, Shengyue Liu, Yongzhou Wang.

**Data curation:** Jiayuan Zhang, Tian Lan.

**Formal analysis:** Yao Peng, Tao Ye, Jiayuan Zhang.

**Funding acquisition:** Shengyue Liu.

**Investigation:** Yao Peng, Tian Lan.

**Methodology:** Yao Peng, Jiayuan Zhang, Tao Ye, Shengyue Liu.

**Project administration:** Shengyue Liu, Yongzhou Wang.

**Resources:** Tian Lan.

**Software:** Yao Peng.

**Supervision:** Yongzhou Wang.

**Visualization:** Yao Peng.

**Writing – original draft:** Yao Peng, Tao Ye.

**Writing – review and editing:** Yao Peng, Yongzhou Wang.
